# Repdigits as sums of three Padovan numbers

**DOI:** 10.1007/s40590-019-00269-9

**Published:** 2019-11-21

**Authors:** Mahadi Ddamulira

**Affiliations:** Institute of Analysis and Number Theory, Graz University of Technology, Kopernikusgasse 24/II, 8010 Graz, Austria

**Keywords:** Padovan number, Repdigit, Linear form in logarithms, Baker’s method, 11B39, 11D45, 11D61, 11J86

## Abstract

Let $$ \{P_{n}\}_{n\ge 0} $$ be the sequence of Padovan numbers defined by $$ P_0=0 $$, $$ P_1 =1=P_2$$, and $$ P_{n+3}= P_{n+1} +P_n$$ for all $$ n\ge 0 $$. In this paper, we find all repdigits in base 10 which can be written as a sum of three Padovan numbers.

## Introduction

Let $\{P_{n}\}_{n\ge 0}$ be the sequence of Padovan numbers given by

$$\begin{aligned} P_0=0, ~P_1= 1, ~P_2=1, \text { and } P_{n+3}= P_{n+1}+P_n \text { for all } n\ge 0. \end{aligned}$$

This is sequence *A*000931 on the On-Line Encyclopedia of Integer Sequences (OEIS) [12]. The first few terms of this sequence are

$$\begin{aligned} \{P_{n}\}_{n\ge 0} = 0, 1, 1, 1, 2, 2, 3, 4, 5, 7, 9, 12, 16, 21, 28, 37, 49, 65, 86, 114, 151, \ldots . \end{aligned}$$

A repdigit is a positive integer *N* that has only one distinct digit when written in base 10. That is, *N* is of the form

1 $$\begin{aligned} N=d\left( \dfrac{10^{\ell }-1}{9}\right) \end{aligned}$$

for some positive integers *d* and $\ell$ with $1\le d\le 9$ and $\ell \ge 2$. The sequence of repdigits is sequence *A*010785 on the OEIS.

## Main Result

In this paper, we study the problem of writing repdigits as sums of three Padovan numbers. More precisely, we completely solve the Diophantine equation:

2 $$\begin{aligned} N=P_{n_1}+P_{n_2}+P_{n_3}=d\left( \dfrac{10^{\ell }-1}{9}\right) , \end{aligned}$$

in non-negative integers $(N, n_1, n_2, n_3, d, \ell )$ with $n_1\ge n_2\ge n_3 \ge 0$, $\ell \ge 2$, and $1\le d\le 9$.

We discard the situations when $n_1=1$ and $n_1=2$ and just count the solutions for $n_1=3$ since $P_1=P_2=P_3 =1$. For the same reasons, we discard the situation when $n_1=4$ and just count the solutions for $n_1=5$ since $P_4=P_5 =2$. Thus, we always assume that $n_1,n_2, n_3 \notin \{1,2,4\}$. Our main result is the following.

### Theorem 1

All non-negative integer solutions $(N, n_1, n_2, n_3, d, \ell )$ with $n_1\ge n_2\ge n_3 \ge 0$, $\ell \ge 2$, and $1\le d\le 9$ to the Diophantine equation (2) arise from

$$\begin{aligned}&N\in \{11, 22, 33, 44, 55, 66, 77, 88, 99, 111, 222, 333, 444, 555,\\&\quad 666, \ 888, 1111, 3333, 7777\}. \end{aligned}$$

This paper serves as a continuation of the results in [3, 6–9, 11]. The method of proof involves the application of Baker’s theory for linear forms in logarithms of algebraic numbers, and the Baker-Davenport reduction procedure. Computations are done with the help of a simple computer program in *Mathematica*.

## Preliminary results

### The Padovan sequence

Here, we recall some important properties of the Padovan sequence $\{P_n\}_{n\ge 0}$. The characteristic equation

$$\begin{aligned} x^3-x-1 = 0 \end{aligned}$$

has roots $\alpha , \beta ,$ and $\gamma = \bar{\beta }$, where

3 $$\begin{aligned} \alpha =\dfrac{r_1+r_2}{6}, \qquad \beta = \dfrac{-(r_1+r_2)+\sqrt{-3}(r_1-r_2)}{12}, \end{aligned}$$

and

4 $$\begin{aligned} r_1=\root 3 \of {108+12\sqrt{69}} \quad \text {and}\quad r_2=\root 3 \of {108-12\sqrt{69}}. \end{aligned}$$

Furthermore, the Binet formula is given by

5 $$\begin{aligned} P_n = a\alpha ^{n}+b\beta ^{n}+c\gamma ^{n} \qquad \text { for all} \quad n\ge 0, \end{aligned}$$

where

6 $$\begin{aligned} \quad a=\dfrac{\alpha +1}{(\alpha -\beta )(\alpha -\gamma )}, \quad b= \dfrac{\beta +1}{(\beta -\alpha )(\beta -\gamma )}, \quad c = \dfrac{\gamma +1}{(\gamma -\alpha )(\gamma -\beta )}={\bar{b}}. \end{aligned}$$

Numerically, the following estimates hold:

7 $$\begin{aligned}&1.32<\alpha<1.33,\nonumber \\&0.86< |\beta |=|\gamma |=\alpha ^{-\frac{1}{2}}< 0.87,\nonumber \\&0.72<a<0.73,\nonumber \\&0.24<|b|=|c|<0.25. \end{aligned}$$

From (3), (4), and (7), it is easy to see that the contribution the complex conjugate roots $\beta$ and $\gamma$, to the right-hand side of (5), is very small. In particular, setting

8 $$\begin{aligned} e(n):=P_n-a\alpha ^{n}=b\beta ^{n}+c\gamma ^{n} \quad \text { it is seen that} \quad |e(n)|< \dfrac{1}{\alpha ^{n/2}}, \end{aligned}$$

holds for all $n\ge 1$. Furthermore, by induction, it can be proved that

9 $$\begin{aligned} \alpha ^{n-3}\le P_n \le \alpha ^{n-1} \quad \text {holds for all }\quad n\ge 1. \end{aligned}$$

### Linear forms in logarithms

Let $\eta$ be an algebraic number of degree *d* with minimal primitive polynomial over the integers:

$$\begin{aligned} a_{0}x^{d}+ a_{1}x^{d-1}+\cdots +a_{d} = a_{0}\prod _{i=1}^{d}(x-\eta ^{(i)}), \end{aligned}$$

where the leading coefficient $a_{0}$ is positive and the $\eta ^{(i)}$’s are the conjugates of $\eta$. Then the *logarithmic height* of $\eta$ is given by

$$\begin{aligned} h(\eta ) := \dfrac{1}{d}\left( \log a_{0} + \sum _{i=1}^{d}\log \left( \max \{|\eta ^{(i)}|, 1\}\right) \right) . \end{aligned}$$

In particular, if $\eta = p/q$ is a rational number with $\gcd (p,q) = 1$ and $q>0$, then $h(\eta ) = \log \max \{|p|, q\}$. The following are some of the properties of the logarithmic height function $h(\cdot )$, which will be used in the next sections of this paper without reference:

10 $$\begin{aligned} h(\eta _1\pm \eta _2)\le & {} h(\eta _1) +h(\eta _2) +\log 2,\nonumber \\ h(\eta _1\eta _2^{\pm 1})\le & {} h(\eta _1) + h(\eta _2),\nonumber \\ h(\eta ^{s})= & {} |s|h(\eta ) \quad (s\in \mathbb {Z}). \end{aligned}$$

For the proofs of (10) and further details, we refer the reader to the book of Bombieri and Gubler [1].

We recall the result of Bugeaud, Mignotte, and Siksek [2, Theorem 9.4, p. 989], which is a modified version of the result of Matveev [10], which is one of our main tools in this paper.

#### Theorem 2

Let $\eta _1,\ldots ,\eta _t$ be positive real algebraic numbers in a real algebraic number field $\mathbb {K} \subset \mathbb {R}$ of degree $D_\mathbb {K}$, $b_1,\ldots ,b_t$ be nonzero integers, and assume that

11 $$\begin{aligned} \varLambda :=\eta _1^{b_1}\cdots \eta _t^{b_t} - 1, \end{aligned}$$

is nonzero. Then

$$\begin{aligned} \log |\varLambda | > -1.4\times 30^{t+3}\times t^{4.5}\times D_\mathbb {K}^{2}(1+\log D_{\mathbb {K}})(1+\log B)A_1\cdots A_t, \end{aligned}$$

where

$$\begin{aligned} B\ge \max \{|b_1|, \ldots , |b_t|\}, \end{aligned}$$

and

$$\begin{aligned} A _i \ge \max \{D_{\mathbb {K}} h(\eta _i), |\log \eta _i|, 0.16\},\qquad {\text {for all}}\qquad i=1,\ldots ,t. \end{aligned}$$

### Reduction procedure

During the calculations, we get upper bounds on our variables which are too large, thus we need to reduce them. To do so, we use some results from the theory of continued fractions.

For the treatment of linear forms homogeneous in two integer variables, we use the well-known classical result in the theory of Diophantine approximation. The following lemma is the criterion of Legendre.

#### Lemma 1

Let $\tau$ be an irrational number, $\frac{p_0}{q_0}, \frac{p_1}{q_1}, \frac{p_2}{q_2}, \ldots$ be all the convergents of the continued fraction expansion of $\tau$ and *M* be a positive integer. Let *N* be a nonnegative integer such that $q_N> M$. Then, putting $a(M):=\max \{a_{i}: i=0, 1, 2, \ldots , N\}$, the inequality:

$$\begin{aligned} \left| \tau - \dfrac{r}{s}\right| > \dfrac{1}{(a(M)+2)s^{2}}, \end{aligned}$$

holds for all pairs (*r*, *s*) of positive integers with $0<s<M$.

For a nonhomogeneous linear form in two integer variables, we use a slight variation of a result due to Dujella and Pethő (see [4, Lemma 5a]). For a real number *X*, we write $\Vert X \Vert := \min \{|X-n|: n\in \mathbb {Z}\}$ for the distance from *X* to the nearest integer.

#### Lemma 2

Let *M* be a positive integer, $\frac{p}{q}$ be a convergent of the continued fraction expansion of the irrational number $\tau$ such that $q>6M$, and $A,B,\mu$ be some real numbers with $A>0$ and $B>1$. Furthermore, let $\varepsilon : = \Vert \mu q \Vert -M\Vert \tau q\Vert$. If $\varepsilon > 0$, then there is no solution to the inequality:

$$\begin{aligned} 0<|u\tau -v+\mu |<AB^{-w}, \end{aligned}$$

in positive integers *u*, *v*, and *w* with

$$\begin{aligned} u\le M \quad {\text {and}}\quad w\ge \dfrac{\log (Aq/\varepsilon )}{\log B}. \end{aligned}$$

Finally, the following Lemma is also useful. It is Lemma 7 in [5].

#### Lemma 3

If $r\geqslant 1$, $H>(4r^2)^r$, and $H>L/(\log L)^r$, then

$$\begin{aligned} L<2^rH(\log H)^r. \end{aligned}$$

## Bounding the variables

We assume that $n_1\ge n_2\ge n_3$. From (2) and (9), we have

$$\begin{aligned} \alpha ^{n_1-3}\le P_{n_1} \le P_{n_1}+P_{n_2}+P_{n_3} = d\left( \dfrac{10^{\ell }-1}{9}\right) \le 10^{\ell } \end{aligned}$$

and

$$\begin{aligned} 10^{\ell -1} \le d\left( \dfrac{10^{\ell }-1}{9}\right) = P_{n_1}+P_{n_2}+P_{n_3} \le 3P_{n_1} < \alpha ^{n_1+3}, \end{aligned}$$

where we use $\alpha ^4 >3$. Thus

$$\begin{aligned} (n_1-3)\dfrac{\log \alpha }{\log 10} \le \ell \quad \text {and}\quad \ell -1 \le (n_1+3)\dfrac{\log \alpha }{\log 10}. \end{aligned}$$

Since $\log \alpha /\log 10 = 0.122123\ldots <1/5$, we can conclude from the above that

12 $$\begin{aligned} (n_1-3)/10< \ell < (n_1+8)/5. \end{aligned}$$

Running a *Mathematica* program in the range $0\le n_3 \le n_2 \le n_1 \le 500$, $1 \le d \le 9$, and $1 \le \ell \le 100$ we obtain only the solutions listed in Theorem 1. From now on, we assume that $n_1> 500$.

By using (8), equation (2) can be written as

13 $$\begin{aligned} a\alpha ^{n_1}+e(n_1)+a\alpha ^{n_2}+e(n_2)+a\alpha ^{n_3}+e(n_3) =d \left( \dfrac{ 10^{\ell }-1}{9}\right) . \end{aligned}$$

We then consider (13) in three different cases as follows.

### Case 1

We have that

$$\begin{aligned} a\alpha ^{n_1}+e(n_1)+a\alpha ^{n_2}+e(n_2)+a\alpha ^{n_3}+e(n_3) -\dfrac{d\cdot 10^{\ell }}{9} = -\dfrac{d}{9}. \end{aligned}$$

This is equivalent to

$$\begin{aligned} a\alpha ^{n_1}- \dfrac{d\cdot 10^{\ell }}{9} = -\dfrac{d}{9} -a(\alpha ^{n_2}+\alpha ^{n_3})- e(n_1)-e(n_2)-e(n_3). \end{aligned}$$

This shows that

$$\begin{aligned} \left| a\alpha ^{n_1}- \dfrac{d\cdot 10^{\ell }}{9}\right|\le & {} \dfrac{d}{9}+ a(\alpha ^{n_2}+\alpha ^{n_3})+|e(n_1)|+|e(n_2)|+|e(n_3)|\\< & {} 1+2a\alpha ^{n_2}+3\alpha ^{-n_3/2}\\< & {} 5a\alpha ^{n_2}, \end{aligned}$$

and so

14 $$\begin{aligned} \left| a\alpha ^{n_1}- \dfrac{d\cdot 10^{\ell }}{9}\right| < 5a\alpha ^{n_2}. \end{aligned}$$

We divide through (14) by $a\alpha ^{n_1}$ to get

$$\begin{aligned} \left| 10^{\ell }\alpha ^{-n_1}\left( \dfrac{d}{9a}\right) -1\right|< & {} 5\alpha ^{n_2-n_1}. \end{aligned}$$

Thus, we have

15 $$\begin{aligned} \left| 10^{\ell }\alpha ^{-n_1}\left( \dfrac{d}{9a}\right) -1\right| < \dfrac{5}{\alpha ^{n_1-n_2}}. \end{aligned}$$

We put

$$\begin{aligned} \varLambda _1:= 10^{\ell }\alpha ^{-n_1}\left( \dfrac{d}{9a}\right) -1. \end{aligned}$$

To apply Theorem 2, we need to check that $\varLambda _1\ne 0$. Suppose that $\varLambda _1=0$, then we have

16 $$\begin{aligned} a\alpha ^{n_1} = \dfrac{10^{\ell }\cdot d}{9}. \end{aligned}$$

To see that this is not true, we consider the $\mathbb {Q}$-automorphism $\sigma$ of the Galois extension $\mathbb {Q}(\alpha , \beta )$ over $\mathbb {Q}$ given by $\sigma (\alpha ):=\beta$ and $\sigma (\beta ):=\alpha$. Now, since $\varLambda _1=0$, we get $\sigma (\varLambda _1)=0$. Thus, conjugating the relation (16) under $\sigma$, and taking absolute values on both sides, we get

$$\begin{aligned} \dfrac{10^{\ell }\cdot d}{9} = |\sigma (a\alpha ^{n_1})|=|b||\beta |^{n_1}< |b|< \dfrac{1}{3}, \end{aligned}$$

which is false for $\ell \ge 2$ and $d \ge 1$. Therefore, $\varLambda _1\ne 0$.

Therefore, we apply Theorem 2 with the data

$$\begin{aligned}&t:=3, \quad \eta _1:=10, \quad \eta _2:=\alpha , \quad \eta _3:=\dfrac{d}{9a},\quad b_1:=\ell , \quad b_2:=-n_1, \quad b_3:=1. \end{aligned}$$

It is a well–known fact that

$$\begin{aligned} a=\dfrac{\alpha (\alpha +1)}{3\alpha ^2-1}, \end{aligned}$$

the minimal polynomial of *a* is $23x^3-23x^2+6x-1$ and has roots *a*, *b*, *c*. Since $|b|=|c|< |a|=a <1$ (by (7)), we get

$$\begin{aligned} h(a)=\dfrac{1}{3}\log 23. \end{aligned}$$

Since $\eta _1, \eta _2, \eta _2\in \mathbb {Q}(\alpha )$, we take the field $\mathbb {K}:= \mathbb {Q}(\alpha )$ with degree $D_{\mathbb {K}}:=3$. Since $\max \{1, \ell , n_1\} \le n_1$, we take $B:=n_1$. Further, the minimal polynomial of $\alpha$ over $\mathbb {Z}$ is $x^3-x-1$ has roots $\alpha , ~ \beta , ~ \gamma$ with $1.32< \alpha < 1.33$ and $|\beta |=|\gamma | <1$. Thus, we can take $h(\alpha ) = \frac{1}{3} \log \alpha$. Similarly, $h(10) = \log 10$ . Also,

$$\begin{aligned} h(\eta _3)\le & {} h(d)+h(9) + h(a) \le 4\log 3+\dfrac{1}{3}\log 23 < 5\log 3 \end{aligned}$$

Thus, we can take $A_1:= 3\log 10$, $A_2:=\log \alpha$ and $A_3:=15\log 3$. So, Theorem 2 tells us that the left-hand side of (15) is bounded below by

$$\begin{aligned} \log |\varLambda _1|> & {} -\,1.4 \times 30^{6}\times 3^{4.5}\times 3^{2}(1+\log 3)(1+\log n_1)(3\log 10)(\log \alpha )(15\log 3)\\> & {} -\,6.16\times 10^{14}\log n_1 \log \alpha . \end{aligned}$$

By comparing the above inequality with the right-hand side of (15) we get that

17 $$\begin{aligned} n_1-n_2 \le 6.18 \times 10^{14}\log n_1. \end{aligned}$$

### Case 2

We have that

$$\begin{aligned} a\alpha ^{n_1}+e(n_1)+a\alpha ^{n_2}+e(n_2) -\dfrac{d\cdot 10^{\ell }}{9} = -\dfrac{d}{9}-a\alpha ^{n_3}-e(n_3). \end{aligned}$$

This is equivalent to

$$\begin{aligned} a(\alpha ^{n_1} +\alpha ^{n_2})- \dfrac{d\cdot 10^{\ell }}{9} = -\dfrac{d}{9} -a\alpha ^{n_3}- e(n_1)-e(n_2)-e(n_3). \end{aligned}$$

Thus, it follows that

$$\begin{aligned} \left| a(\alpha ^{n_1}+\alpha ^{n_2})- \dfrac{d\cdot 10^{\ell }}{9}\right|\le & {} \dfrac{d}{9}+ a\alpha ^{n_3}+|e(n_1)|+|e(n_2)|+|e(n_3)|\\< & {} 1+a\alpha ^{n_3}+3\alpha ^{-n_3/2}\\< & {} 3a\alpha ^{n_3}, \end{aligned}$$

and so

18 $$\begin{aligned} \left| a(\alpha ^{n_1}+\alpha ^{n_2})- \dfrac{d\cdot 10^{\ell }}{9}\right| < 3a\alpha ^{n_3}. \end{aligned}$$

We divide through (14) by $a(\alpha ^{n_1}+\alpha ^{n_2})$ to get

$$\begin{aligned} \left| 10^{\ell }\alpha ^{-n_2}\left( \dfrac{d}{9a(1+\alpha ^{n_1-n_2})}\right) -1\right|< & {} \dfrac{3\alpha ^{n_3-n_2}}{1+\alpha ^{n_1-n_2}}. \end{aligned}$$

This means that

19 $$\begin{aligned} \left| 10^{\ell }\alpha ^{-n_2}\left( \dfrac{d}{9a(1+\alpha ^{n_1-n_2})}\right) -1\right| < \dfrac{3}{\alpha ^{n_2-n_3}}. \end{aligned}$$

We put

$$\begin{aligned} \varLambda _2:= 10^{\ell }\alpha ^{-n_2}\left( \dfrac{d}{9a(1+\alpha ^{n_1-n_2})}\right) -1. \end{aligned}$$

As before, to apply Theorem 2, we need to check that $\varLambda _2\ne 0$. Suppose that $\varLambda _2=0$, then we have

20 $$\begin{aligned} a(\alpha ^{n_1}+\alpha ^{n_2}) = \dfrac{10^{\ell }\cdot d}{9}. \end{aligned}$$

To see that this is not true, we again consider the $\mathbb {Q}$-automorphism $\sigma$ of the Galois extension $\mathbb {Q}(\alpha , \beta )$ over $\mathbb {Q}$ given by $\sigma (\alpha ):=\beta$ and $\sigma (\beta ):=\alpha$. Now, since $\varLambda _2=0$, we get $\sigma (\varLambda _2)=0$. Thus, conjugating the relation (20) under $\sigma$, and taking absolute values on both sides, we get

$$\begin{aligned} \dfrac{10^{\ell }\cdot d}{9} = |\sigma (a(\alpha ^{n_1}+\alpha ^{n_2}))|=|b|(|\beta |^{n_1}+|\beta |^{n_2})< 2|b|< \dfrac{2}{3}, \end{aligned}$$

which is false for $\ell \ge 2$ and $d \ge 1$. Therefore, $\varLambda _2\ne 0$.

Therefore, we apply Theorem 2 with the data

$$\begin{aligned} t:= & {} 3, \quad \eta _1:=10, \quad \eta _2:=\alpha , \quad \eta _3:=\dfrac{d}{9a(1+\alpha ^{n_1-n_2})},\quad \\ b_1:= & {} \ell , \quad b_2:=-n_2, \quad b_3:=1. \end{aligned}$$

Since $\eta _1, \eta _2, \eta _2\in \mathbb {Q}(\alpha )$, we take the field $\mathbb {K}:= \mathbb {Q}(\alpha )$ with degree $D_{\mathbb {K}}:=3$. Since $\max \{1, \ell , n_2\} \le n_1$, we take $B:=n_1$. Further,

$$\begin{aligned} h(\eta _3)\le & {} h(d)+h(9) + h(a) + h(1+\alpha ^{n_1-n_2})\\ {}\le & {} 4\log 3+\dfrac{1}{3}\log 23 +(n_1-n_2)\log \alpha + \log 2\\ {}< & {} 1.77\times 10^{14}\log n_1 \quad (\text {by }(17)). \end{aligned}$$

Thus, we can take $A_1:= 3\log 10$, $A_2:=\log \alpha$ and $A_3:=5.31\times 10^{14}\log n_1$. So, Theorem 2 tells us that the left-hand side of (19) is bounded below by

$$\begin{aligned} \log |\varLambda _2|> & {} -1.4 \times 30^{6}\times 3^{4.5}\times 3^{2}(1+\log 3)(1+\log n_1)(3\log 10)(\log \alpha )(5.31\\&\times \, 10^{14}\log n_1)\\> & {} -\,1.98\times 10^{28}(\log n_1)^2 \log \alpha . \end{aligned}$$

By comparing the above inequality with the right-hand side of (19), we get that

21 $$\begin{aligned} n_2-n_3 \le 2\times 10^{28}(\log n_1)^2. \end{aligned}$$

### Case 3

We have that

$$\begin{aligned} a\alpha ^{n_1}+e(n_1)+a\alpha ^{n_2}+e(n_2) + a\alpha ^{n_3}+e(n_3) -\dfrac{d\cdot 10^{\ell }}{9} = -\dfrac{d}{9}. \end{aligned}$$

This is equivalent to

$$\begin{aligned} a(\alpha ^{n_1} +\alpha ^{n_2}+\alpha ^{n_3})- \dfrac{d\cdot 10^{\ell }}{9} = -\dfrac{d}{9}- e(n_1)-e(n_2)-e(n_3). \end{aligned}$$

Thus, we have

$$\begin{aligned} \left| a(\alpha ^{n_1} +\alpha ^{n_2}+\alpha ^{n_3})- \dfrac{d\cdot 10^{\ell }}{9}\right|\le & {} \dfrac{d}{9}+|e(n_1)|+|e(n_2)|+|e(n_3)|\\< & {} 1+3\alpha ^{-n_3/2}< 3, \end{aligned}$$

and so

22 $$\begin{aligned} \left| a(\alpha ^{n_1} +\alpha ^{n_2}+\alpha ^{n_3})- \dfrac{d\cdot 10^{\ell }}{9}\right| < 3. \end{aligned}$$

We divide through (14) by $a(\alpha ^{n_1} +\alpha ^{n_2}+\alpha ^{n_3})$ to get

$$\begin{aligned} \left| 10^{\ell }\alpha ^{-n_3}\left( \dfrac{d}{9a(1+\alpha ^{n_1-n_3}+\alpha ^{n_2-n_3})}\right) -1\right|< & {} \dfrac{3\alpha ^{-n_1}}{(1+\alpha ^{n_2-n_1}+\alpha ^{n_3-n_1})}. \end{aligned}$$

Thus, it follows that

23 $$\begin{aligned} \left| 10^{\ell }\alpha ^{-n_3}\left( \dfrac{d}{9a(1+\alpha ^{n_1-n_3}+\alpha ^{n_2-n_3})}\right) -1\right| < \dfrac{5}{\alpha ^{n_1}}. \end{aligned}$$

We put

$$\begin{aligned} \varLambda _3:= 10^{\ell }\alpha ^{-n_3}\left( \dfrac{d}{9a(1+\alpha ^{n_1-n_3}+\alpha ^{n_2-n_3})}\right) -1. \end{aligned}$$

As before, in order to apply Theorem 2 we need to check that $\varLambda _3\ne 0$. Suppose that $\varLambda _3=0$, then we have

24 $$\begin{aligned} a(\alpha ^{n_1}+\alpha ^{n_2}+\alpha ^{n_3}) = \dfrac{10^{\ell }\cdot d}{9}. \end{aligned}$$

To see that this is not true, we again consider the $\mathbb {Q}$-automorphism $\sigma$ of the Galois extension $\mathbb {Q}(\alpha , \beta )$ over $\mathbb {Q}$ given by $\sigma (\alpha ):=\beta$ and $\sigma (\beta ):=\alpha$. Now, since $\varLambda _3=0$, we get $\sigma (\varLambda _3)=0$. Thus, conjugating the relation (24) under $\sigma$, and taking absolute values on both sides, we get

$$\begin{aligned} \dfrac{10^{\ell }\cdot d}{9} = |\sigma (a(\alpha ^{n_1}+\alpha ^{n_2}+\alpha ^{n_3}))|=|b|(|\beta |^{n_1}+|\beta |^{n_2}+|\beta |^{n_3})< 3|b|< 1, \end{aligned}$$

which is false for $\ell \ge 2$ and $d \ge 1$. Therefore, $\varLambda _2\ne 0$.

Therefore, we apply Theorem 2 with the data:

$$\begin{aligned}&t:=3, \quad \eta _1:=10, \quad \eta _2:=\alpha , \quad \eta _3:=\dfrac{d}{9a(1+\alpha ^{n_1-n_3}+\alpha ^{n_2-n_3})},\\&b_1:=\ell , \quad b_2:=-n_3, \quad b_3:=1. \end{aligned}$$

Since $\eta _1, \eta _2, \eta _2\in \mathbb {Q}(\alpha )$, we take the field $\mathbb {K}:= \mathbb {Q}(\alpha )$ with degree $D_{\mathbb {K}}:=3$. Since $\max \{1, \ell , n_3\} \le n_1$, we take $B:=n_1$. Furthermore

$$\begin{aligned} h(\eta _3)\le & {} h(d)+h(9) + h(a) + h(1+\alpha ^{n_1-n_3}+\alpha ^{n_2-n_3})\\ {}\le & {} 4\log 3+\dfrac{1}{3}\log 23 +((n_1-n_3)+ (n_2-n_3))\log \alpha + 2\log 2\\< & {} 6.6\log 3+ ((n_1-n_2)+2(n_2-n_3))\log \alpha \\< & {} 1.72\times 10^{28}(\log n_1)^2 \quad (\text {by }(17)\hbox { and }(21)). \end{aligned}$$

Thus, we can take $A_1:= 3\log 10$, $A_2:=\log \alpha$, and $A_3:=5.16\times 10^{28}(\log n_1)^2$. So, Theorem 2 tells us that the left-hand side of (23) is bounded below by

$$\begin{aligned} \log |\varLambda _3|> & {} -1.4 \times 30^{6}\times 3^{4.5}\times 3^{2}(1+\log 3)(1+\log n_1)(3\log 10)\\&\times \, (\log \alpha )(5.16\times 10^{28}(\log n_1)^2)\\> & {} -1.92\times 10^{42}(\log n_1)^3 \log \alpha . \end{aligned}$$

By comparing the above inequality with the right-hand side of (19), we get that

25 $$\begin{aligned} n_1\le 1.94\times 10^{42}(\log n_1)^3. \end{aligned}$$

Now, we apply Lemma 3 on the above inequality (25) with the data: $r:=3, ~ H:=1.94\times 10^{42}$, and $L:=n_1$. We obtain that $n_1 < 2.7\times 10^{48}$. We now record what we have proved.

#### Lemma 4

Let $(N, n_1, n_2, n_3, d, \ell )$ be the nonnegative integer solutions to the Diophantine equation (2) with $n_1\ge n_2 \ge n_3 \ge 0$, $1\le d\le 9$, and $\ell \ge 2$. Then, we have

$$\begin{aligned} \ell< n_1 < 3\times 10^{48}. \end{aligned}$$

## Reducing the bounds

The bounds obtained in Lemma 4 are too large to carry out meaningful computations on the computer. Thus, we need to reduce these bounds. To do so, we return to (15), (19), and (23) and apply Lemma 2 via the following procedure.

First, we put

$$\begin{aligned} \varGamma _1:=\ell \log 10 - n_1\log \alpha +\log \left( \frac{d}{9a}\right) , \quad 1\le d\le 9. \end{aligned}$$

For technical reasons, we assume that $n_1-n_2 \ge 20$ and go to (15). Note that $e^{\varGamma _1}-1= \varLambda _1 \ne 0$. Thus, $\varGamma _1\ne 0$. If $\varGamma _1< 0$, then

$$\begin{aligned} 0< |\varGamma _1|< e^{|\varGamma _1|}-1 = |\varLambda _1| < \dfrac{5}{\alpha ^{n_1-n_2}}. \end{aligned}$$

If $\varGamma _1> 0$, then we have that $|e^{\varGamma _1}-1| <1/2$. Hence $e^{\varGamma _1}<2$. Thus, we get that

$$\begin{aligned} 0< \varGamma _1< e^{\varGamma _1}-1 = e^{\varGamma _1}|\varLambda _1| < \dfrac{10}{\alpha ^{n_1-n_2}}. \end{aligned}$$

Therefore, in both cases, we have that

$$\begin{aligned} 0< |\varGamma _1|=\left| \ell \log 10 - n_1\log \alpha +\log \left( \frac{d}{9a}\right) \right| < \dfrac{10}{\alpha ^{n_1-n_2}}. \end{aligned}$$

Dividing through the above inequality by $\log \alpha$, we get

26 $$\begin{aligned} 0< \left| \ell \dfrac{\log 10}{\log \alpha } - n_1 + \dfrac{\log (d/(9a))}{\log \alpha }\right| < \dfrac{10}{\alpha ^{n_1-n_2}\log \alpha }. \end{aligned}$$

If we put

$$\begin{aligned} \tau :=\dfrac{\log 10}{\log \alpha } \quad \text {and} \quad \mu (d):=\dfrac{\log (d/(9a))}{\log \alpha }, \quad 1\le d\le 9, \end{aligned}$$

we can rewrite (26) as

27 $$\begin{aligned} 0< \left| \ell \tau - n_1 + \mu (d)\right| < 36\cdot \alpha ^{-(n_1-n_2)}. \end{aligned}$$

We now apply Lemma 2 on (27). We put $M:=3\times 10^{48}$. A quick computer search in *Mathematica* reveals that the convergent

$$\begin{aligned} \dfrac{p_{106}}{q_{106}}=\dfrac{177652856036642165557187989663314255133456297895465}{21695574963444524513646677911090250505443859600601} \end{aligned}$$

of $\tau$ is such that $q_{106}> 6M$ and $\varepsilon (d) \ge 0.0129487>0$. Therefore, with $A:=36$ and $B:=\alpha$, we calculated each value of $\log (36q_{106}/\varepsilon (d))/\log \alpha$ and found that all of them are at most 432. Thus, we have that $n_1-n_2 \le 432.$ In the case $n_1-n_2 <20$, we have $n_1-n_2<20<432$. Thus, $n_1-n_2 \le 432$ holds in both cases.

Next, we put

$$\begin{aligned} \varGamma _2:=\ell \log 10 - n_2\log \alpha +\log \left( \frac{d}{9a(1+\alpha ^{n_1-n_2})}\right) , \quad 1\le d\le 9. \end{aligned}$$

For technical reasons, as before we assume that $n_2-n_3 \ge 20$ and go to (19). Note that $e^{\varGamma _2}-1= \varLambda _2 \ne 0$. Thus, $\varGamma _2\ne 0$. If $\varGamma _2< 0$, then

$$\begin{aligned} 0< |\varGamma _2|< e^{|\varGamma _2|}-1 = |\varLambda _2| < \dfrac{3}{\alpha ^{n_2-n_3}}. \end{aligned}$$

If $\varGamma _2> 0$, then we have that $|e^{\varGamma _2}-1| <1/2$. Hence $e^{\varGamma _2}<2$. Thus, we get that

$$\begin{aligned} 0< \varGamma _2< e^{\varGamma _2}-1 = e^{\varGamma _2}|\varLambda _2| < \dfrac{6}{\alpha ^{n_2-n_3}}. \end{aligned}$$

Therefore, in both cases, we have that

$$\begin{aligned} 0< |\varGamma _2|=\left| \ell \log 10 - n_1\log \alpha +\log \left( \frac{d}{9a(1+\alpha ^{n_1-n_2})}\right) \right| < \dfrac{6}{\alpha ^{n_2-n_3}}. \end{aligned}$$

Dividing through the above inequality by $\log \alpha$, we get

28 $$\begin{aligned} 0< \left| \ell \dfrac{\log 10}{\log \alpha } - n_2 + \dfrac{\log (d/(9a(1+\alpha ^{n_1-n_2})))}{\log \alpha }\right| < \dfrac{6}{\alpha ^{n_2-n_3}\log \alpha }. \end{aligned}$$

We put

$$\begin{aligned} \tau :=\dfrac{\log 10}{\log \alpha } \quad \text {and} \quad \mu (d,k):=\dfrac{\log (d/(9a(1+\alpha ^{k}))}{\log \alpha }, \quad 1\le d\le 9, \quad 1\le k \le 432, \end{aligned}$$

where $k:=n_1-n_2$. We can rewrite (28) as

29 $$\begin{aligned} 0< \left| \ell \tau - n_2 + \mu (d,k)\right| < 22\cdot \alpha ^{-(n_2-n_3)}. \end{aligned}$$

We now apply Lemma 2 on (29). We put $M:=3\times 10^{48}$. A quick computer search in *Mathematica* reveals that the 106-th convergent of $\tau$ is such that $q_{106}> 6M$ and $\varepsilon (d,k) \ge 0.000134829>0$ for all $1\le d\le 9$ and $1\le k \le 432$ except for the case $\varepsilon (9,11)$, which is always negative. Therefore, with $A:=22$ and $B:=\alpha$ we calculated each value of $\log (22q_{106}/\varepsilon (d,k))/\log \alpha$ and found that all of them are at most 446. Thus, we have that $n_2-n_3 \le 446.$

The problem in the case of $\varepsilon (9,11)$ is due to the fact that

30 $$\begin{aligned} \dfrac{1}{\alpha ^{9}}= \dfrac{2\alpha ^{3}+1}{\alpha (\alpha +1)(\alpha ^{11}+1)}. \end{aligned}$$

Thus, if we consider the identity (30), the inequality (28) becomes

31 $$\begin{aligned} 0< \left| \tau - \dfrac{(n_2+9)}{\ell }\right| < \dfrac{6}{\alpha ^{n_2-n_3}\ell \log \alpha }. \end{aligned}$$

In this case, we apply the classical result from Diophantine approximation given in Lemma 1. We assume that $n_2-n_3$ is so large that the right-hand side of the inequality in (31) is smaller than $1/(2\ell ^2)$. This certainly holds if

32 $$\begin{aligned} \alpha ^{n_2-n_3}>12\ell /\log \alpha . \end{aligned}$$

Since $\ell< n_1< 3\times 10^{48}$, it follows that the last inequality (32) holds provided that $n_2-n_3\ge 415$, which we now assume. In this case $r/s:=(n_2+9)/\ell$ is a convergent of the continued fraction of $\tau : = \log 10 / \log \alpha$ and $\ell < 3\times 10^{48}$. We are now set to apply Lemma 1.

We write $\tau : =[a_0; a_1, a_2, a_3, \ldots ] = [8; 5, 3, 3, 1, 5, 1, 8, 4, 6, 1, 4, 1, 1, 1, 9, 1, 4, 4, 9, \ldots ]$ for the continued fraction expansion of $\tau$ and $p_k/q_k$ for the $k-$th convergent. We get that $r/s=p_j/q_j$ for some $j\le 106$. Furthermore, putting $a(M):=\max \{a_j: j=0,1, \ldots , 106\}$, we get $a(M):=564$. By Lemma 1, we get

$$\begin{aligned} \dfrac{1}{566\ell ^2}=\dfrac{1}{(a(M)+2)\ell ^{2}}\le \left| \tau -\dfrac{r}{s}\right| < \dfrac{6}{\alpha ^{n_2-n_3}\ell \log \alpha }, \end{aligned}$$

which gives

$$\begin{aligned} \alpha ^{n_2-n_3}< \dfrac{566\times 6\ell }{\log \alpha } < \dfrac{566\times 6 \times 3\times 10^{48}}{\log \alpha }. \end{aligned}$$

This implies that $n_2-n_3\le 435$. Thus, in both cases we have that $n_2-n_3\le 446$. In the case $n_2-n_3 < 20$, we get that $n_2-n_3<20< 446$. Thus, $n_2-n_3\le 446$ holds in all cases.

Finally, we put

$$\begin{aligned} \varGamma _3:=\ell \log 10 - n_3\log \alpha +\log \left( \frac{d}{9a(1+\alpha ^{n_1-n_3}+\alpha ^{n_2-n_3})}\right) , \quad 1\le d\le 9. \end{aligned}$$

We use the assumption that $n_1>500$ and go to (23). Note that $e^{\varGamma _3}-1= \varLambda _3 \ne 0$. Thus, $\varGamma _3\ne 0$. If $\varGamma _3< 0$, then

$$\begin{aligned} 0< |\varGamma _3|< e^{|\varGamma _3|}-1 = |\varLambda _3| < \dfrac{5}{\alpha ^{n_1}}. \end{aligned}$$

If $\varGamma _3> 0$, then we have that $|e^{\varGamma _3}-1| <1/2$. Hence $e^{\varGamma _3}<2$. Thus, we get that

$$\begin{aligned} 0< \varGamma _3< e^{\varGamma _3}-1 = e^{\varGamma _3}|\varLambda _3| < \dfrac{10}{\alpha ^{n_1}}. \end{aligned}$$

Therefore, in both cases, we have that

$$\begin{aligned} 0< |\varGamma _3|=\left| \ell \log 10 - n_3\log \alpha +\log \left( \frac{d}{9a(1+\alpha ^{n_1-n_2}+\alpha ^{n_2-n_3})}\right) \right| < \dfrac{10}{\alpha ^{n_1}}. \end{aligned}$$

Dividing through the above inequality by $\log \alpha$, we get

33 $$\begin{aligned} 0< \left| \ell \dfrac{\log 10}{\log \alpha } - n_3 + \dfrac{\log (d/(9a(1+\alpha ^{n_1-n_3}+\alpha ^{n_2-n_3}))}{\log \alpha }\right| < \dfrac{10}{\alpha ^{n_1}\log \alpha }. \end{aligned}$$

We put

$$\begin{aligned} \tau :=\dfrac{\log 10}{\log \alpha } \quad \text {and} \quad \mu (d,k,s):=\dfrac{\log \left( d/(9a(1+\alpha ^{k}+\alpha ^{s}))\right) }{\log \alpha }, \quad 1\le d\le 9, \end{aligned}$$

where $1\le k:=n_1-n_3 =(n_1-n_2)+(n_2-n_3) \le 878$ and $1\le s:=n_2-n_3 \le 446$. We can rewrite (33) as

34 $$\begin{aligned} 0< \left| \ell \tau - n_3 + \mu (d,k,s)\right| < 36\cdot \alpha ^{-n_1}. \end{aligned}$$

We now apply Lemma 2 on (34). We put $M:=3\times 10^{48}$. A quick computer search in *Mathematica* reveals that the 106-th convergent of $\tau$ is such that $q_{106}> 6M$ and $\varepsilon (d,k,s) \ge 0.000125>0$. Therefore, with $A:=36$ and $B:=\alpha$ we calculated each value of $\log (36q_{106}/\varepsilon (d,k,s))/\log \alpha$ and found that all of them are at most 485. Thus, $n_1\le 485$. This contradicts our assumption that $n_1> 500$. Hence, Theorem 1 holds. $\square$
